# Change in Trust in US Government Health Agencies for Cancer Information in the COVID-19 Era

**DOI:** 10.1001/jamanetworkopen.2024.23744

**Published:** 2024-07-22

**Authors:** Onyema G. Chido-Amajuoyi, Rajesh Talluri, Henry K. Onyeaka, Itunu Sokale, Gideon T. Dosunmu, Noelle LoConte, Sanjay Shete

**Affiliations:** 1 Department of Epidemiology, The University of Texas MD Anderson Cancer Center, Houston; 2 Department of Internal Medicine, Texas A&M College of Medicine/Christus Health, Longview; 3 Department of Data Science, The University of Mississippi Medical Center, Jackson; 4 Department of Psychiatry, Harvard Medical School, Boston, Massachusetts; 5 Department of Psychiatry, Massachusetts General Hospital, Boston; 6 Department of Medicine, Baylor College of Medicine, Houston, Texas; 7 Section of Hematology-Oncology, Department of Medicine, University of Chicago, Chicago, Illinois; 8 Division of Hematology, Medical Oncology and Palliative Care, Department of Medicine, University of Wisconsin School of Medicine and Public Health, Madison; 9 Carbone Cancer Center, Madison, Wisconsin; 10 Department of Biostatistics, The University of Texas MD Anderson Cancer Center, Houston; 11 Division of Cancer Prevention and Population Sciences, The University of Texas MD Anderson Cancer Center, Houston

## Abstract

This cross-sectional study assesses changes in levels of public trust in US government health agencies providing cancer information.

## Introduction

While public trust in the US government has been historically suboptimal, government health agencies have until recently enjoyed high levels of public trust.^1,2^ A study using national data from 2005 to 2015 revealed that the agencies were the second most trusted source of health information after physicians.^3^ However, recent studies revealed changes to this trust dynamic,^2,4^ especially since the COVID-19 pandemic.^2,5^ A study of population-level trust in general health recommendations from several health agencies found that high-level trust was low for the Centers for Disease Control and Prevention (37%) and the National Institutes of Health (33%).^5^ Several US government health agencies provide cancer information to the public; thus, trust in information from these agencies is critical for adoption of their recommendations. A recent study^6^ found that more than 20% of US adults had little or no trust in governmental health organizations for cancer-related information. Hence, we examined changes in public trust in cancer information from government health agencies as well as the sociodemographic correlates of this change.

## Methods

We examined data from a national representative survey of noninstitutionalized civilian US adults: the US Health Information National Trends Survey (HINTS) 5 cycle 4 (2020) and HINTS 6 (2022). The response rate for HINTS 5 cycle 4 was 36.7% and 28.1% for HINTS 6. In accordance with 45 CFR §46, this cross-sectional study was exempt from institutional review board approval because the data are publicly available. We followed the STROBE reporting guideline.

The primary outcome was trust in government health agencies providing cancer information (eMethods in Supplement 1). Weighted prevalence and corresponding 95% CIs were calculated to estimate the level of trust in government health agencies providing cancer information for both study years within the overall study sample. Trust was also assessed by respondents’ sociodemographic characteristics. Participant race and ethnicity were self-reported. Statistical analyses were performed using the survey package in R, version 4.3.1 (R Project for Statistical Computing).

## Results

The study included 3582 respondents (mean [SD] age, 47.8 [17.9] years; 51.0% female and 49.0% male [weighted percentages]) in 2020 and 5979 respondents (mean [SD] age, 48.4 [17.9] years; 50.7% female and 49.3% male [weighted percentages]) in 2022 (Table 1). Weighted percentages of Hispanic, non-Hispanic Black, and non-Hispanic White respondents were 16.4%, 10.8%, and 64.8%, respectively, in 2020 and 16.5%, 11.0%, and 61.7% in 2022.

**Table 1.** Descriptive Characteristics of the Study Population

Characteristic	2020 Respondents		2022 Respondents	
	No.	Weighted No. (%)	No.	Weighted No. (%)
No.	3582	241 050 744.9 (100)	5979	249 349 949.0 (100)
Age, y				
18-34	477	65 318 346.8 (27.1)	931	65 764 876.4 (26.4)
35-49	682	62 634 645.3 (26.0)	1219	64 368 894.9 (25.8)
≥50	2322	113 097 752.8 (46.9)	3743	119 216 177.7 (47.8)
Sex				
Female	2041	123 016 606.2 (51.0)	3356	126 417 958.5 (50.7)
Male	1464	118 034 138.8 (49.0	2231	122 931 990.4 (49.3)
Race and ethnicity^a^				
Hispanic	533	39 548 945.6 (16.4)	919	41 175 435.1 (16.5)
Non-Hispanic Asian	148	11 517 554.8 (4.8)	276	14 000 621.9 (5.6)
Non-Hispanic Black	433	25 980 595.2 (10.8)	849	27 383 280 (11.0)
Non-Hispanic White	2072	156 143 779.8 (64.8)	3126	153837903.6 (61.7)
Non-Hispanic other^b^	113	7 859 869.5 (3.3)	180	12 952 708.4 (5.2)
Residence				
Rural	401	29 756 881.7 (12.3)	785	31019661.7 (12.4)
Urban	3181	211 293 863.2 (87.7)	5194	218 330 287.3 (87.6)
Income, $				
0-9999	216	12 187 022.2 (5.1)	441	17 698 764.1 (7.1)
10 000-34 999	862	52 140 147.3 (21.6)	1368	47 664 947.7 (19.1)
35 000-74 999	1099	73 210 829.5 (30.4)	1806	73 556 735.4 (29.5)
≥75 000	1390	103 512 745.9 (42.9)	2351	110 429 501.8 (44.3)
Educational level				
<High school	216	17 687 696.7 (7.3)	313	14 698 675.5 (5.9)
High school graduate	614	51 822 157.3 (21.5)	988	53 310 207.4 (21.4)
Some college education	1020	96 031 094.2 (39.8)	1619	97 849 539.6 (39.2)
College graduate or higher	1623	75 509 796.8 (31.3)	2672	83 491 526.4 (33.5)

^a^ Race and ethnicity data were collected for the US Health Information National Trends Survey to allow subgroup-specific analysis of the data by race and ethnicity.

^b^ Includes non-Hispanic American Indian or Alaska Native, non-Hispanic Native Hawaiian or Other Pacific Islander, and non-Hispanic multiple races.

In 2022, a significant decrease in public trust in government agencies providing cancer information was noted, with 70.1% (95% CI, 68.1%-72.0%) of respondents expressing trust compared with 77.8% (95% CI, 75.4%-80.0%) in 2020. Trust also significantly decreased among respondents aged 18 to 34 years (69.6% [95% CI, 63.5%-75.2%] in 2022 vs 82.7% [95% CI, 75.9%-87.8%] in 2020) (Table 2).

**Table 2.** Trust in Government Agencies Providing Cancer Information

	Trust among 2020 respondents, weighted % (95% CI)	Trust among 2022 respondents, weighted % (95% CI)
Overall trust	77.8 (75.4-80.0)	70.1 (68.1-72.0)
Age, y		
18-34	82.7 (75.9-87.8)	69.6 (63.5-75.2)
35-49	76.7 (71.7-81.1)	70.0 (65.7-73.9)
≥50	75.7 (72.6-78.6)	70.6 (68.6-72.6)
Sex		
Female	77.9 (75.0-80.6)	72.8 (70.0-75.4)
Male	77.7 (73.4-81.4)	67.6 (64.4-70.7)
Race and ethnicity^a^		
Hispanic	82.2 (74.8-87.8)	71.5 (64.8-77.3)
Non-Hispanic Asian	88.8 (78.9-94.4)	82.2 (66.4-91.5)
Non-Hispanic Black	74.4 (66.6-80.9)	72.4 (64.7-79.0)
Non-Hispanic White	77.8 (74.7-80.6)	70.0 (67.7-72.2)
Non-Hispanic other^b^	65.0 (48.2-78.8)	58.5 (41.3-73.9)
Residence		
Rural	74.8 (67.8-80.7)	64.4 (59.7-68.8)
Urban	78.2 (75.6-80.6)	70.9 (68.8-72.9)
Income, $		
0-9999	73.8 (64.5-81.4)	69.4 (59.8-77.5)
10 000-34 999	77.7 (73.2-81.6)	67.5 (62.6-72.1)
35 000-74 999	73.6 (66.9-79.3)	70.9 (66.9-74.5)
≥75 000	81.1 (77.3-84.3)	71.6 (68.5-74.6)
Education		
<High school	73.4 (60.3-83.4)	64.6 (54.8-73.4)
High school graduate	66.6 (60.6-72.1)	61.3 (56.7-65.7)
Some college education	79.9 (75.2-83.9)	68.5 (64.6-72.2)
≥College graduate	83.6 (80.4-86.4)	79.8 (77.4-81.9)

^a^ Race and ethnicity data were collected for the US Health Information National Trends Survey to allow subgroup-specific analysis of the data by race and ethnicity.

^b^ Includes non-Hispanic American Indian or Alaska Native, non-Hispanic Native Hawaiian or Other Pacific Islander, and non-Hispanic multiple races.

Among respondents with some college education, public trust in US health agencies decreased to 68.5% (95% CI, 64.6%-72.2%) in 2022 from 79.9% (95% CI, 75.2%-83.9%) in 2020. Similarly, a significant decrease was observed among Non-Hispanic White respondents (70.0% [95% CI, 67.7%-72.2%] in 2022 vs 77.8% [95% CI, 74.7%-80.6%] in 2020). Additionally, among respondents with an income of $75 000 or more, trust decreased to 71.6% (95% CI, 68.5%-74.6%) in 2022 from 81.1% (95% CI, 77.3%-84.3%) in 2020. Significant decreases were also observed among male respondents and urban residents (Table 2).

## Discussion

Findings of this cross-sectional study have important implications for cancer prevention, treatment, and outcomes given that trust is critical to the adoption of cancer health recommendations from these agencies. In turn, the ability of federal health agencies to implement public health interventions effectively is dependent on public trust. Therefore, targeted interventions at the population-level that further understanding and address factors contributing to the decrease in trust in US health agencies are essential.

Study limitations include possible low-response bias and the cross-sectional design of the survey, which precluded our ability to make causal inferences. Moreover, since respondents were not followed up longitudinally, we were unable to examine if there were shifts in individual respondents’ trust over time.

## Notes

Supplement 1.
eMethods.

Supplement 2.
Data Sharing Statement
