# Supplementary material for: Change in Trust in US Government Health Agencies for Cancer Information in the COVID-19 Era
Source: JAMA Netw Open. 2024 Jul 22;7(7):e2423744. doi: 10.1001/jamanetworkopen.2024.23744 (PMC11265138; doi:10.1001/jamanetworkopen.2024.23744)
Supplement: Supplement 1. — eMethods. [file jamanetwopen-e2423744-s001.pdf]

Supplemental Online Content

Ghido-Amajuoyi OG, Talluri R, Onyeaka HK, et al. Trust in US government health agencies for cancer information in the COVID-19 era. *JAMA Netw Open*. 2024;7(7):e2423744. doi:10.1001/jamanetworkopen.2024.23744

eMethods.

This supplemental material has been provided by the authors to give readers additional information about their work.

© 2024 Chido-Amajuoyi OG et al. *JAMA Network Open.*

eMethods

Study Data and Population

We examined data from the National Cancer Institute's Health Information National Trends Survey (HINTS) 5 Cycle 4 (survey/data collection period February through June 2020), and HINTS 6 (survey/data collection period March through November 2022.) HINTS is a nationally representative survey of noninstitutionalized civilian US adults. The sampling design for HINTS 5 Cycle 4 and HINTS 6 employed a two-stage design. In the first stage, a stratified sample of addresses was selected from a file of residential addresses. In the second stage, one adult was selected within each sampled household. The sampling frame consisted of a database of addresses used by Marketing Systems Group (MSG) to provide random samples of addresses. The survey response rate for HINTS 5 Cycle 4 was 36.7% and 28.1% for HINTS 6. Of note, 96.8% and 95% of the respondents responded to the trust question in 2022 and 2020, respectively. Data from HINTS are publicly available and de-identified. Therefore, our study is exempt from institutional review board review per the Common Rule (45 CFR §46). This study followed the STROBE reporting guideline.

Study Variables

Outcome Measure

The primary focus of our study was to investigate self-reported trust in government health agencies for cancer information. Participants were asked to express their level of trust in the information about cancer originating from "Government Health agencies." The response options were categorized into four levels, including: "Not at all," "a little," "some," and "a lot." We further categorized responses into two groups: Trust (including responses of "some" and "a lot") and Lack of Trust (including responses of "Not at all "and "a little").

Participant Characteristics

The study outcome was examined by stratifying the key sociodemographic characteristics of respondents which were age, sex, race and ethnicity, educational level, household income, and rural-urban residence. Age was grouped into 3 categories: 18 to 34 years, 35 to 49 years, and 50 years and older. Race and ethnicity was categorized as non-Hispanic white, non-Hispanic black, Hispanic and Non-Hispanic Asian and Non-Hispanic Other. Education levels were grouped into 4 categories: Less than high school, high school graduate, some college (a combination of post–high school training other than college and some college training), and college graduate or postgraduate. The residence was defined using the US Department of Agriculture's 2013 Rural-Urban Continuum Codes. Codes 1 to 3 were designated as urban, while 4 to 9 were categorized as rural. Household annual income was categorized as less than $9,999, $10,000 to $35,000 to $49,999, $50,000 to $74,999, and $75,000 or more.

Data Analysis

© 2024 Chido-Amajuoyi OG et al. *JAMA Network Open.*

To ensure the representativeness of our findings, survey weights were applied to the study data as stipulated by HINTS, aiming to mirror the demographics of the entire United States population accurately. The weighted prevalence and corresponding 95% confidence intervals were calculated to estimate the level of trust in government health organizations for cancer information for both study years within the overall study sample.

Trust in government health organizations for cancer information was assessed within distinct sociodemographic subgroups to discern variations influenced by age, education, race, income, and rural-urban residence. Race, a vital component of our sociodemographic assessment, was self-reported by study participants. The statistical significance was conservatively established using non-overlapping 95% confidence intervals of proportions for the years 2020 and 2022. Statistical computations and analyses were executed in R 4.3.1 using the survey package.

© 2024 Chido-Amajuoyi OG et al. *JAMA Network Open.*
