# Supplementary material for: Change in Trust in US Government Health Agencies for Cancer Information in the COVID-19 Era
Source: JAMA Netw Open. 2024 Jul 22;7(7):e2423744. doi: 10.1001/jamanetworkopen.2024.23744 (PMC11265138; doi:10.1001/jamanetworkopen.2024.23744)
Supplement: Supplement 2. — Data Sharing Statement [file jamanetwopen-e2423744-s002.pdf]

Data Sharing Statement

Chido-Amajuoyi. Trust in US Government Health Agencies for Cancer Information in the COVID-19 Era. *JAMA Netw Open*. Published July 22, 2024.
doi:10.1001/jamanetworkopen.2024.23744

Data
Data available: No
